# Impact of Low-Dose CT Radiation on Gene Expression and DNA Integrity

**DOI:** 10.3390/ijms262411869

**Published:** 2025-12-09

**Authors:** Nikolai Schmid, Vadim Gorte, Michael Akers, Niklas Verloh, Michael Haimerl, Christian Stroszczynski, Harry Scherthan, Timo Orben, Samantha Stewart, Laura Kubitscheck, Hanns Leonhard Kaatsch, Matthias Port, Michael Abend, Patrick Ostheim

**Affiliations:** 1 Bundeswehr Institute of Radiobiology affiliated to Ulm University, Neuherbergstr. 11, 80937 Munich, Germany; 2 Department of Radiology, University Hospital Regensburg, Franz-Josef-Strauß-Allee 11, 93053 Regensburg, Germany; 3 Department of Radiology, University Hospital Erlangen, Ulmenweg 18, 91054 Erlangen, Germany; 4 Department of Diagnostic and Interventional Radiology, Borromean Sisters Hospital Trier, Medical Campus Trier of the University Medical Center Mainz, Feldstraße 16, 54290 Trier, Germany; 5 Department of Radiology, Klinikum Würzburg Mitte, Salvatorstr. 7, 97070 Würzburg, Germany; 6 Department of Radiology and Neuroradiology, Bundeswehr Central Hospital, Rübenacher Str. 170, 56072 Koblenz, Germany

**Keywords:** low-dose radiation exposure, biomarker, *FDXR*, *DDB2*, *AEN*, *EDA2R*, gene expression, CT, γ-H2AX, DNA double-strand breaks

## Abstract

Computed tomography (CT) is a major source of low-dose ionizing radiation exposure in medical imaging. Risk assessment at this dose level is difficult and relies on the hypothetical linear no-threshold model. To address the response to such low doses in patients undergoing CT scans, we examined radiation-induced alterations at the transcriptomic and DNA damage levels in peripheral blood cells. Peripheral whole blood of 60 patients was collected before and after CT. Post-CT samples were obtained 4–6 h after scan (n = 28, in vivo incubation) or alternatively immediately after the CT scan, followed by ex vivo incubation (n = 32). The gene expression of known radiation-responsive genes (n = 9) was quantified using qRT-PCR. DNA double-strand breaks (DSB) were assessed in 12 patients through microscopic γ-H2AX + 53BP1 DSB focus staining. The mean dose–length product (DLP) across all scans was 561.9 ± 384.6 mGy·cm. Significant differences in the median differential gene expression (DGE) were detected between in vivo and ex vivo incubation conditions, implicating that ex vivo incubation masked the true effect in low-dose settings. The median DGE of in vivo-incubated samples showed a significant upregulation of *EDA2R*, *MIR34AHG*, *PHLDA3*, *DDB2*, *FDXR*, and *AEN* (*p* ranging from <0.001 to 0.041). In vivo, we observed a linear dose-dependent upregulation for several genes and an explained variance of 0.66 and 0.56 for *AEN* and *FDXR*, respectively. DSB focus analysis revealed a slight, non-significant increase in the average DSB damage post-exposure, at a mean DLP of 321.0 mGy·cm. Our findings demonstrate that transcriptional biomarkers are sensitive indicators of low-dose radiation exposure in medical imaging and could prove themselves as clinically applicable biodosimetry tools. Furthermore, the results underscore the need for dose optimization.

## 1. Introduction

The effects elicited by ionizing radiation have been a subject of extensive research for decades, with its biological effects well-documented, especially at higher exposure levels [1]. The linear no-threshold (LNT) model, widely used in radiation protection, posits a direct correlation between radiation doses and long-term adverse health effects, suggesting that there is no safe exposure level [2]. While severe health effects such as acute radiation syndrome occur at higher doses (>1 Sv) [3], the impact of low-dose exposures (<100 mSv) remains largely speculative. In developed countries, medical procedures represent a main source of radiation exposure for the general public [4]. Notably, in Germany, computed tomography (CT) scans contribute about 60% of the medical radiation dose, while fluoroscopic procedures account for 20% [5]. Despite technological advancements reducing individual exposure, the increasing frequency of diagnostic imaging accounts for significant collective doses.

Radiation can induce cellular changes even at low doses, which may potentially contribute to tumor formation over time through genetic alterations due to misrepair. The challenge lies in establishing causal relationships, not only for irradiation-induced tumor development but also for non-cancerous conditions such as cardiovascular diseases that may manifest decades later [6,7,8].

This research aims to enhance risk assessments for low-dose radiation exposures by investigating the underlying biological mechanisms that remain largely unknown. While many observed clinically relevant radiation effects stem from high-dose exposures (≥1 Sv), medical imaging rarely exceeds whole-body effective doses of 20 mSv per examination [9].

In biodosimetry assessments of such low-dose exposures, traditional chromosome-based methods, like the dicentric chromosome assay, have proven inadequate, albeit these are accurate in higher-dose ranges [10,11,12]. Consequently, molecular methods focusing on radiation-induced alterations in DNA, RNA, and proteins are gaining prominence. Advanced technologies such as next-generation sequencing (NGS) and quantitative real-time PCR (qRT-PCR) enable the high-throughput analysis of radiation-induced gene expression changes [13,14,15]. In this investigation, we used qRT-PCR to detect transcriptomic changes and the γ-H2AX DSB focus assay, capable of detecting DNA double-strand breaks (DSB) at absorbed doses below 10 mGy [16,17,18].

This study seeks to validate changes in candidate genes’ expressions associated with low-dose radiation exposure, as identified in previous research [19,20], and compares these results with the formation of γ-H2AX DSB-indicating foci after CT exposure.

## 2. Results

### 2.1. Patient Characteristics

In total, 61 patients participated in the study. One patient was unexpectedly not available for a second blood draw for the gene expression assay; he therefore was excluded from further analysis regarding gene expression, but this patient provided blood for the γ-H2AX focus assay. Out of the 60 patients, 39 (65%) were male and 21 (35%) were female. The mean age of the participants was 65.2 ± 14.4 years. The mean DLP over all 60 patients was 561.9 ± 384.6 mGy·cm, corresponding to an estimated effective dose of 8.3 ± 5.8 mSv. The mean DLP reported for the 12 patients who provided additional samples for the γ-H2AX focus assay was 321.0 ± 149.3 mGy·cm, with a respective effective dose estimate of 4.3 ± 2.4 mSv (Table 1).

### 2.2. Gene Expression Analysis

All genes (*AEN*, *BAX*, *DDB2*, *EDA2R*, *FDXR*, *MIR34AHG*, *PHLDA3*, *POU2AF1*, *WNT3*) were detected in all samples.

**Figure 1:**
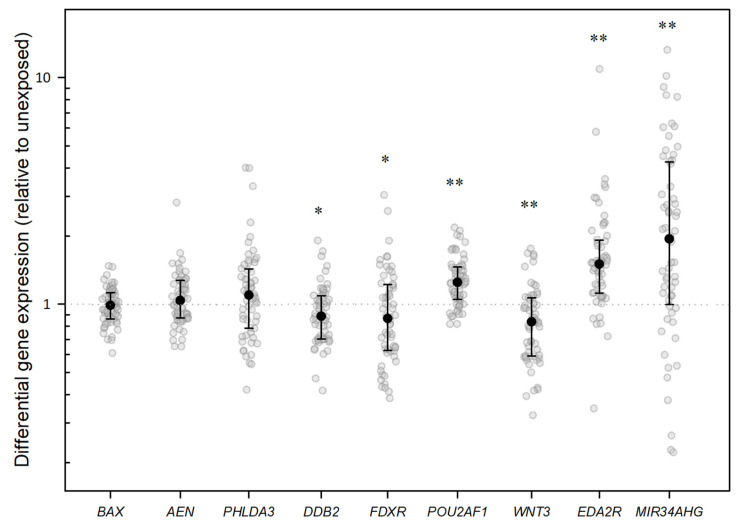
Jitter plots illustrate the distribution of differential gene expression (DGE) after CT scan for the indicated genes in the analysis of all samples (in vivo and ex vivo combined). The graphs show DGE normalized with *PUM1* and relative to the unexposed in vivo blood sample. Black dots represent the median, with the whiskers representing the interquartile range (25th–75th percentiles) and the dotted line the baseline gene expression. Statistical analysis was performed in log2 transformed data. A single asterisk indicates a gene that is significantly differentially expressed compared with the control at a significance level of *p* < 0.05, and two asterisks denote a significance level of *p* < 0.001.

**Figure 2:**
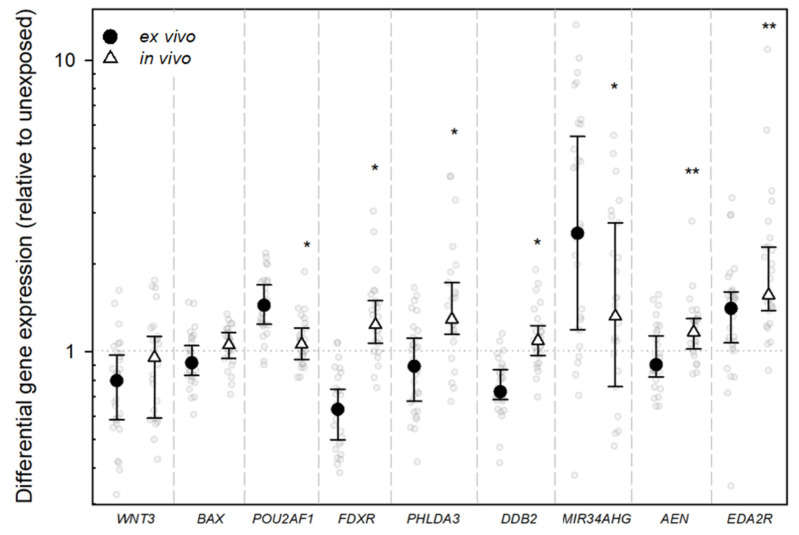
Jitter plots illustrate the distribution of differential gene expression (DGE) after CT scan for the indicated genes, with comparison between DGE of in vivo- and ex vivo-incubated samples. The graphs show DGE normalized with *PUM1* and relative to the unexposed in vivo blood sample. Black dots represent the median value, with the whiskers representing the interquartile range (25th–75th percentiles) and the dotted line the baseline gene expression. Statistical analysis was performed in log2 transformed data. A single asterisk indicates a gene that is significantly differentially expressed compared with the control at a significance level of *p* < 0.05, and two asterisks denote a significance level of *p* < 0.001. Significance levels are solely displayed for the in vivo-incubated samples.

Are CT irradiation-induced gene expression changes detectable? When all samples were initially analyzed, combined (in vivo and ex vivo), highly significant (*p* ≤ 0.001) median DGE changes were detectable after CT exposure for *EDA2R*, *MIR34AHG*, and *WNT3. EDA2R* and *MIR34AHG* were upregulated and *WNT3* was downregulated. For *DDB2* and *FDXR*, we observed a slight downregulation (*p* ≤ 0.05), and for *POU2AF1* we observed a significant upregulation (*p* ≤ 0.001, Figure 1, Appendix A.1). When analyzing in vivo-incubated samples, *DDB2*, *FDXR*, *AEN*, and *PHLDA3* also showed a significant upregulation relative to unexposed samples (*p* ≤ 0.001–0.041). However, *WNT3* was no longer differentially expressed (*p* = 0.302), and *POU2AF1* appeared slightly upregulated (*p* = 0.049; Figure 2).

**Figure 3:**
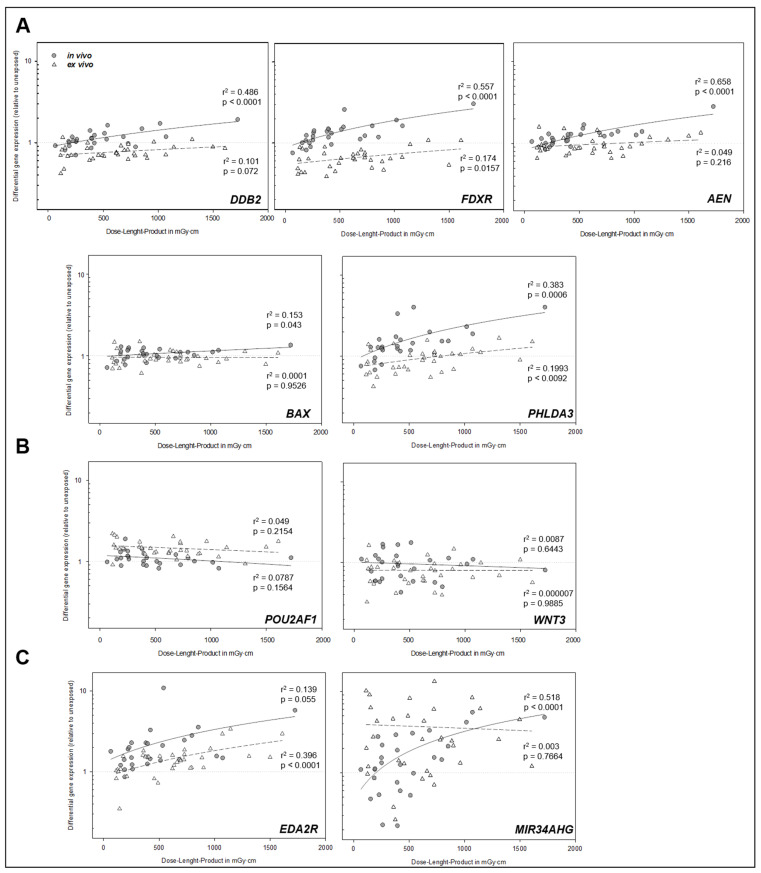
Linear regression analysis between dose–length product (DLP) and differential gene expression levels for each investigated gene. Dark circles represent in vivo-incubated samples, while white triangles represent ex vivo-incubated samples. The solid line indicates the linear regression curve fit for in vivo samples, and the dashed line for ex vivo samples. Regression statistics (r^2^ and *p*-values) are shown for each condition. In the first row (**A**), genes exhibiting a strong and highly significant relationship between DLP and DGE (especially in vivo) are visualized. (**B**) displays genes with borderline significance or low r^2^ values in vivo. (**C**) shows genes for which no apparent relationship between DLP and DGE was observed in vivo and ex vivo.

Are there GE differences between in vivo and ex vivo incubation? For all genes of interest except *WNT3*, we found a significant difference in the median DGE when comparing the in vivo- with the ex vivo-incubated samples (*p* ≤ 0.001–0.03). Apart from *MIR34AHG*, a slightly greater degree of anticipated DGE was observed across all genes in the in vivo-incubated samples (Table 2, Figure 3). In seven of the nine genes, a reduction in gene expression was observed in the ex vivo samples at very low dose ranges compared to the in vivo samples, but *FDXR*, *PHLDA3*, and *EDA2R* also showed a tendency of upregulation ex vivo at higher DLP levels (Figure 3).

**Figure 4:**
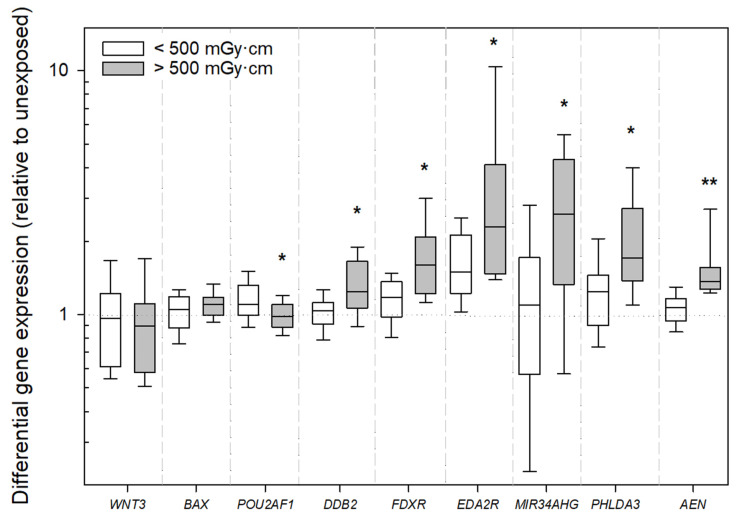
Boxplot showing differential gene expression (relative to unexposed samples) for the analyzed genes, stratified by dose–length product (DLP) for the in vivo-incubated samples. Samples exposed to <500 mGy·cm are represented by white boxes, and those exposed to >500 mGy·cm by gray boxes. A single asterisk indicates a significant difference between the groups at a significance level of *p* < 0.05, and two asterisks denote a significance level of *p* < 0.001.

Are radiation-induced gene expression changes dose-dependent? An analysis of dose-dependent differences in DGE was performed using linear regression analysis. The results demonstrated, for the in vivo samples, a significant linear relationship between DLP and DGE for several genes. Specifically, we found a highly significant linear relationship for *AEN*, *FDXR*, *DDB2*, and *PHLDA3* (all *p* < 0.0001, Figure 4). For *BAX*, a lower but still significant r^2^ of 0.15 was observed (*p* = 0.043), while *EDA2R* showed a borderline-significant association with an r^2^ of 0.14 (*p* = 0.055, Figure 3). All of these genes were upregulated after the CT scan. For the ex vivo-incubated samples, the relationship between DLP and DGE was generally weaker, corresponding to a several-fold-lower r^2^ (exemplary 3.2 times for *FDXR*, Figure 3). For *FDXR* and *PHLDA3*, a modest but statistically significant linear association (*p* = 0.009–0.016) was observed. In contrast, no statistically significant linear relationships were observed for *DDB2*, *AEN*, or *BAX* in the ex vivo samples. Notably, only *EDA2R* demonstrated a more pronounced linear relationship in the ex vivo-incubated samples (*p* < 0.0001, Figure 4). Additionally, stratifying patients with in vivo incubation into two groups based on a DLP cutoff of 500 mGy·cm (for approximately the same sized groups) revealed significant differences, with a higher upregulation of *DDB2*, *FDXR*, *AEN*, *EDA2R*, *MIR34AHG*, and *PHLDA3* in individuals receiving more than 500 mGy·cm compared to those exposed to doses below this cutoff (Figure 4).

Are there sex-dependent differences in gene expression? Only *WNT3* showed significant differences between males and females, with females showing a stronger downregulation relative to male patients when considering all patients (*p* = 0.02; Table 2). When the analysis was restricted to in vivo samples, no significant differences between the two genders could be observed.

### 2.3. DNA Double-Strand Break Analysis

**Figure 5:**
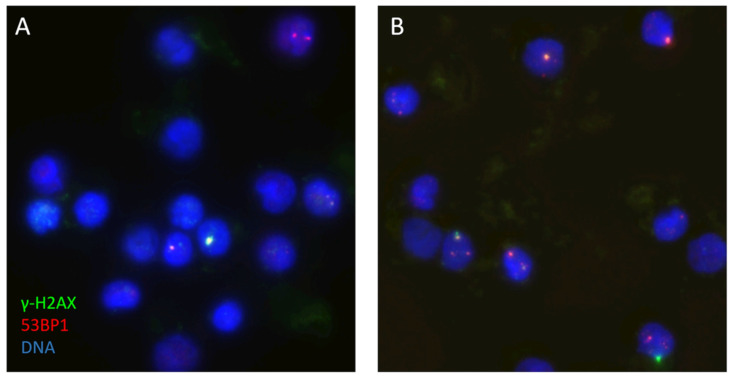
Image fields of the γ-H2AX (green) + 53BP1 (red) DSB focus assay of patient No. 45, showing the DSB foci before (**A**) and after (**B**) a CT scan of the abdomen and pelvis with a DLP of 509 mGy·cm. The DNA of the PBMC nuclei is stained blue with DAPI.

In a subset of twelve patients who provided additional samples for the DSB focus assay, γ-H2AX + 53BP1 DSB-indicating foci were analyzed, which show a 1/1 correlation in patient PBMCs [21]. The mean foci-per-cell count increased from 0.60 ± 0.25 in unexposed samples to 0.70 ± 0.29 following CT exposure, indicating the average induction of 0.1 ± 0.15 radiation-induced foci per cell. However, this slight IR-induced increase was not statistically significant (*p* = 0.37, Figure 5, Appendix A.2).

## 3. Discussion

A large part of the dose contribution of medical ionizing radiation exposure to the population in developed countries is caused by the rising number of CT examinations [22]. Therefore, investigating the biological effects of low-dose irradiation is gaining importance.

A comparative analysis of in vivo and ex vivo incubation revealed substantial differences in DGE across all investigated genes, except *WNT3*, despite matched incubation periods. In vivo conditions exhibited a stronger concordance with the expected transcriptional patterns based on previous work [19,20,23,24]. This highlights the relevance of the native cellular milieu in regulating transcriptional dynamics and the incapacity of ex vivo models to accurately replicate physiological responses. The observed divergence could be the consequence of sample handling abnormalities during ex vivo incubation, such as slight temperature variations, or it could represent physiological processes such paracrine signaling and cell communication [25,26,27,28]. The modest effect sizes brought on by low-dose radiation may be the cause of the mismatch between the ex vivo model and the anticipated DGE patterns seen in our investigation, which could result in an overrepresentation of these minor in vitro effects.

In the initial analysis of all samples, *EDA2R* and *MIR34AHG* exhibited a highly significant upregulation (*p* < 0.001), and *WNT3* exhibited a significant downregulation (*p* < 0.001), all in expected directions [19,20,23,24]. Contrasting with the established radiation-altered gene expression, *DDB2* and *FDXR* appeared downregulated and *POU2AF1* upregulated in the initial analysis, when all samples (in vivo- and ex vivo-incubated) were considered [19,23,24]. However, an analysis of in vivo conditions revealed the significant upregulation of *DDB2* and *FDXR*, so the initial results are most likely attributed the aforementioned ex vivo effect (Figure 3 and Figure 4). Additionally, *AEN* and *PHLDA3* showed an upregulation after in vivo incubation (*p* < 0.001–0.003), implying that these genes play a role in the cellular response to radiation [19,20,29].

Our findings demonstrate that ionizing radiation-induced gene expression changes exhibit a dose-dependent pattern in vivo. Specifically, we observed a linear relationship between DLP and DGE for *DDB2*, *FDXR*, *AEN*, *MIR34AHG*, and *PHLDA3*, as well as a weaker linear relationship for *BAX*. A borderline-significant linear relationship was also found for *EDA2R*.

Notably, this linear association was stronger relative to ex vivo and for most genes restricted to in vivo-incubated samples (Figure 3).

For the in vivo-incubated samples, stratification with a DLP cutoff of 500 mGy·cm revealed the significant upregulation of *DDB2*, *FDXR*, *POU2AF1*, *AEN*, *EDA2R*, *MIR34AHG*, and *PHLDA3* in individuals exposed to higher doses. These observations confirm that ionizing radiation triggers specific transcriptional programs that scale with damage severity [20,30,31], while also confirming the potential of DLP as a representative surrogate for X-ray-induced DNA damage [32].

Recent ex vivo studies have validated *EDA2R* gene expression changes as a highly sensitive biomarker, demonstrating a significant upregulation at doses as low as 2.6 mGy and a linear increase in expression up to 49.7 mGy. While *FDXR* also responds to ionizing radiation, it requires higher doses for significant upregulation compared to *EDA2R* [33]. Another recent study also showed the dose-dependent upregulation *DDB2*, *FDXR*, *AEN*, *EDA2R*, *MIR34AHG*, and *PHLDA3* after CT exposure using next-generation sequencing [20]. These findings could now be validated with our work using qRT-PCR. The upregulation of these genes is closely linked to the p53 pathway, a central regulator of the cellular DNA damage response [20,34,35]. The observed dose-dependent transcriptional responses underscore that CT-induced DNA damage is quantifiable at the molecular level and activates functional gene networks involved in repair and apoptosis [36].

Sex-dependent differences were minimal, with only *WNT3* showing significant variation (*p* = 0.02) when in vivo and ex vivo samples were combined. In vivo, no sex-dependent differences could be observed. This aligns with the limited evidence for sex-dependent ionizing radiation responses in clinical biodosimetry, though larger cohorts are needed to validate this observation [37].

The γ-H2AX focus assay, being a well-established surrogate assay for assessing DNA double-strand breaks as hallmark of radiation-induced dsDNA damage [38], showed a dose-dependent response to CT-induced DSBs, with significant DSB foci increases observed at doses exceeding a DLP of 500 mGy·cm, with the induced DSBs rapidly declining over time [16,39]. In the subset of twelve CT-exposed patients, γ-H2AX + 53BP1 DSB foci analysis revealed a slight but non-significant increase in DSB-marking foci (mean increase: 0.1 foci/cell, *p* = 0.37). This small increase contrasts with the significant transcriptional responses, suggesting that CT-induced DNA damage may fall below the detection threshold of the γ-H2AX/53BP1 assay under these experimental conditions, or that the statistical power was too low (see below). Previous studies indicate that γ-H2AX foci quantification is highly sensitive to radiation quality, dose rate, and post-exposure timing. The observed mean foci count (0.60 ± 0.25 unexposed vs. 0.70 ± 0.29 post-exposure) aligns with the expected range for low-dose CT exposures but highlights methodological limitations. The assay’s resolution may be insufficient for detecting subtle DSB increases from lower diagnostic CT doses, as in our sub-cohort, particularly given inter-individual variability in DNA repair efficiency [40]. Earlier research demonstrated an increase in DSB numbers following a CT scan carried out in vitro [19,33]. Our findings suggest that, at the low doses encountered in diagnostic CT scans, the induced average DSB foci values may be affected by post-exposure repair during the 30 min post-scan time until fixation. In agreement, a post-CT time-dependent decline of CT-induced foci values has been observed to be in the range of 40% [39]. Moreover, a considerable inter-individual variation among patients will also reduce statistical power. Our findings agree with prior research, which failed to reveal a significant DSB induction after low-dose chest CT with an effective dose of 1.5 mSv [41]. Furthermore, the insignificant increase in DSB frequency in this investigation may be attributed to the exposure conditions during the CT scan, which in most cases equals partial body irradiation. The circulation of blood cells through the body leads to a short traversal time through the radiation field and volume, and subsequent mixing with non-irradiated blood cells may have lowered the average foci-per-cell values [42,43]. In support, a partial body-irradiated volume effect leading to reduced foci counts after chest CT relative to chest–abdominal–pelvic CT has been reported by Rothkamm et al. [39].

The discordance between significant DGE changes and a non-significant increase in the average CT-induced DSB foci numbers suggests that transcriptional biomarkers (e.g., *FDXR*, *AEN*, *MIR34AHG*) may offer superior sensitivity and a larger informative time window for low-dose CT biodosimetry compared to direct DSB focus quantification. These results mirror previous findings that gene expression signatures can detect radiation responses at doses below those reliably measured by γ-H2AX [33]. Additionally, the in vivo irradiation conditions and time of sample collection could have led to a higher sensitivity of gene expression because of the aforementioned processes like radiation-induced paracrine signaling.

Still, this study has several limitations. The number of patients evaluated for γ-H2AX foci was small (n = 12), which could have potentially limited the statistical power to detect subtle increases in DSB damage. Heterogeneity in patient characteristics, CT scan protocols, and different health conditions of patients may have influenced the results. Additionally, ex vivo incubation may not fully replicate the physiological conditions of in vivo exposure in low-dose settings. Our findings indicate that in vivo models have a higher sensitivity and are therefore preferable to ex vivo models in medical imaging studies. Utilizing ex vivo models tends to yield a lower explained variance, which may mask potential associations. Moreover, gene expression changes do not necessarily indicate a higher risk of harmful health consequences like cancer or non-cancerous conditions like cardiovascular diseases, although they provide a sensitive method for identifying cellular responses to ionizing radiation. Since these GE molecular endpoints are markers of cellular stress and reaction rather than absolute predictions of long-term injury, risk assessment based just on transcriptional alterations needs careful extrapolation. Further studies with larger cohorts are required to validate our findings and assess the long-term clinical relevance of early low-dose radiation-induced transcriptional responses.

A particular strength of this investigation is the combination of molecular (qRT-PCR) and cellular (γ-H2AX foci) endpoints in a clinical in vivo setting with patient samples. The study further benefits from its comparative in vivo vs. ex vivo design, offering unique insights into the influence of physiological contexts on radiation responses.

## 4. Materials and Methods

### 4.1. CT Imaging

CT images were acquired by using a 3rd-generation CT scanner. Dose was reported as dose–length product (DLP). Computed Tomography Dose Index (CTDI_Vol_) values were recorded but not used for dose stratification, as DLP (mGy·cm) provides a more comprehensive measure of patient exposure for biological correlation studies [32,44]. Using conversion factors (k), which are specific to distinct body locations, an estimate of the effective doses was calculated [45].

### 4.2. Sample Selection

**Figure 6:**
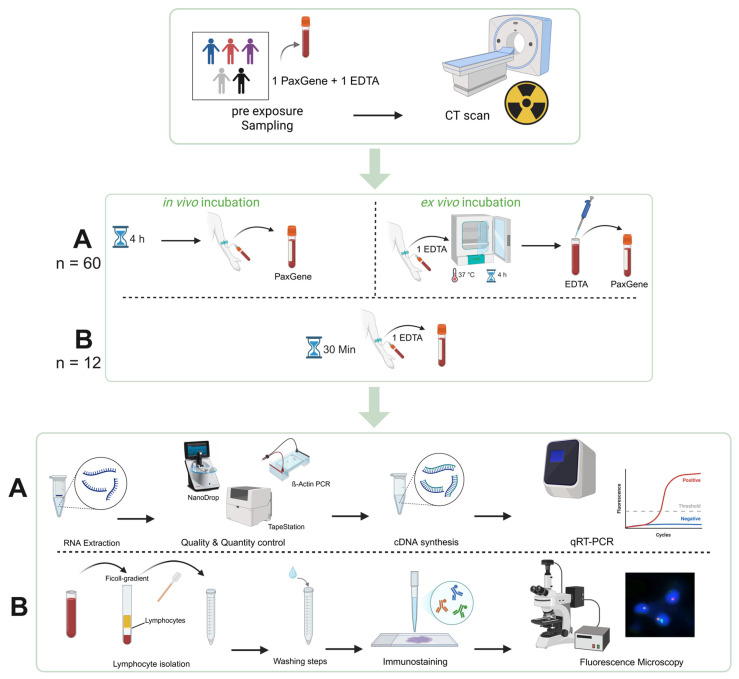
Visualization of the experimental design. Peripheral whole blood samples of 60 patients were collected before and after CT scans. The second blood draw was performed four to six hours after the scan for in vivo incubation. Ex vivo-incubated samples (EDTA tubes) were obtained immediately after the CT scan, followed by incubation at 37 °C for four to six hours (section A). Twelve patients provided additional samples for the γ-H2AX focus assay. These samples were collected 30 min after the CT scan (section B). The samples were then further processed and analyzed in the laboratory according to our protocols. Created in BioRender. RadBio, G. (2025) https://BioRender.com/yc30cpk.

Blood samples were collected pre-exposure directly into PaxGene^®^ Blood RNA tubes (Becton, Dickinson and Company, Franklin Lakes, NJ, USA) and either 4–6 h post-CT scan (in vivo incubation) directly into PaxGene^®^ tubes or immediately after the scan into EDTA tubes and then transferred into PaxGene^®^ tubes after 4 h of ex vivo incubation at 37 °C (Figure 6). Blood samples for DSB foci analysis were collected 30 min after CT scan; peripheral blood mononuclear cells (PBMC) were isolated by Ficoll centrifugation, fixed in 70% Ethanol, and stored at −20 °C until further processing [17]. The tubes were securely packed, transported under strict temperature-controlled conditions, and arrived frozen at our laboratory. All samples (2 per patient, totaling 120 specimens, plus 2 additional samples from 12 patients, totaling 24 specimens for the γ-H2AX assay) were included in this study.

### 4.3. RNA Extraction

Samples were thawed and centrifuged, after which the supernatant was removed. The resulting cell pellets were resuspended in buffers supplemented with proteinase K. RNA extraction was carried out semi-automatically using the QIAsymphony^®^ SP system (QIAGEN, Hilden, Germany) along with the QIAsymphony^®^ Blood RNA Kit (QIAGEN, Hilden, Germany). Throughout the extraction process, RNA was purified via sequential washing and enzymatic digestion steps, utilizing both DNase I and proteinase K. Magnetic beads coated with RNA-binding silica facilitated the isolation. The purified RNA was then eluted in 80 µL of BR5 buffer, incubated at 65 °C for five minutes, and subsequently stored at −20 °C.

### 4.4. Quantity and Quality Control

RNA concentrations were measured using a NanoDrop^®^ spectrophotometer (QIAGEN, Hilden, Germany). The quality of the RNA was evaluated through automated electrophoretic integrity analysis with the 4200 TapeStation System (Agilent Technologies, Santa Clara, CA, USA), which provided RNA integrity number (RIN) values. All samples satisfied the established quality requirements and were subsequently included in the qRT-PCR analysis. Initially, beta-Actin PCR analysis revealed DNA contamination in fifteen samples. These samples underwent manual DNA digestion, after which they were reanalyzed to verify the successful removal of DNA contamination.

### 4.5. Quantitative Real-Time Reverse Transcription Polymerase Chain Reaction (qRT-PCR)

For each sample, 1 µg of total RNA was reverse-transcribed into cDNA using the High-Capacity cDNA Reverse Transcription Kit (Applied Biosystems™, Life Technologies, Darmstadt, Germany). Equal quantities of cDNA were mixed with TaqMan™ Universal PCR Master Mix (Applied Biosystems™, Thermo Fisher Scientific, Waltham, MA, USA) and gene-specific TaqMan™ (Thermo Fisher Scientific, Waltham, MA, USA) assays for *PUM1* (Hs00206469_m1), *AEN* (Hs00224322_m1), *BAX* (Hs00180269_m1), *DDB2* (Hs00172068_m1), *FDXR* (Hs01031617_m1), *EDA2R* (Hs00939736_m1), *MIR34AHG* (Hs04333908_s1), *PHLDA3* (Hs00385313_m1), *POU2AF1* (Hs01573371_m1), and *WNT3* (Hs00902257_m1). qRT-PCR was performed using the QuantStudio™ 12K OA Real-Time PCR System (Thermo Fisher Scientific Inc., Waltham, MA, USA), where each gene was measured in duplicate. Following the implementation of standard operating procedures in our laboratory in 2008, when the Bundeswehr Institute of Radiobiology received DIN EN ISO 9001/2008 [46] certification, all technical procedures were carried out in compliance with these guidelines. The raw cycle threshold (Ct) values were normalized to *PUM1*. Differential gene expression (DGE) was determined using the −ΔΔCt method [47], with the unexposed in vivo sample from the same patient serving as the calibrator ($DGE\;=\;2^{-∆∆{\bar{Ct}}_{preexp}}$).

### 4.6. Immunostaining and DNA DSB Focus Analysis

DNA double-strand break-indicating foci were analyzed through immunofluorescence staining of the DSB damage and repair markers γ-H2AX and 53BP1 [48]. Co-localizing γ-H2AX + 53BP1 foci were counted by an experienced investigator (H.S.) as 100 well-separated and morphologically intact PBMC nuclei per sample using a Zeiss Axio Imager.Z2 fluorescence microscope (Zeiss, Altlussheim, Germany) equipped with a red and green double band pass filter. Radiation-induced foci (RIF) were calculated by subtracting the average DSB foci per cell values of respective control samples before irradiation from the average DSB foci values after irradiation. A RIF value of zero signifies completion of DNA repair.

### 4.7. Statistical Analysis

Mean/median values and standard deviations (SDs) were calculated using Excel (Microsoft, Redmond, WA, USA). Gene expression data were log2-transformed to enable the symmetric comparison of GE before and after irradiation. Differences from zero were assessed using a one-sample *t*-test or Wilcoxon signed-rank test, when applicable, using SigmaPlot (Version 15, Jandel Scientific, Erkrath, Germany). Linear regression analysis was performed to assess the relationship between variables. The resulting regression line and statistical parameters were generated automatically by the software. T-tests or, when the data were not normally distributed, Wilcoxon Rank-Sum tests were used where appropriate. A *p*-value ≤ 0.05 indicated statistical significance.

## 5. Conclusions

Transcriptional biomarkers demonstrated sensitive responses in CT-exposed patients. The γ-H2AX DSB focus assay results suggest that, at the lower spectrum of radiation doses applied in medical imaging, significant increases in IR-induced DNA double-strand breaks are difficult to reveal in this experimental setting.

Our results support the concept that transcriptional biomarkers provide sensitive markers of very-low-dose ionizing radiation exposure. Since DLP can be used to estimate X-ray-induced damage, these discoveries may have useful ramifications for radiation safety and customized risk assessment in medical imaging. This highlights the importance of dose optimization in medical imaging, emphasizing the importance of maintaining radiation exposure as low as reasonably achievable (ALARA) while ensuring diagnostic efficacy.

## Figures and Tables

**Table 1:** Descriptive data of the study population show the number of patients per category, mean values with standard deviation (SD), and minimum (Min) as well as maximum (Max) values.

Categories		N (%)	Mean ± SD	Min	Max
Age [years]		60 (100%)	65.2 ± 14.4	28.0	91.0

Gender	Male	39 (65%)			
	Female	21 (35%)			
DLP [mGy·cm]	All patients	60 (100%)	561.9 ± 384.6	67.0	1725.0
	γ-H2AX group	12 (20%)	321.0 ± 149.3	115.0	622.0
Effective dose estimate [mSv]	All patients	60 (100%)	8.3 ± 5.8	0.9	24.2
	γ-H2AX group	12 (20%)	4.3 ± 2.4	1.3	9.3

**Table 2:** Differences in DGE after CT scan considering different groups of patients. Groups are sorted by incubation method (in vivo or ex vivo) or their gender. Significant findings are highlighted in bold.

Gene of Interest	Incubation			Gender		
	In Vivo n = 27	Ex Vivo n = 33		Male n = 39	Female n = 21	
	Median	Median	*p*-Value	Median	Median	*p*-Value
**DGE Relative to Unexposed**						
*DDB2*	1.09	0.73	**<0.001**			
				0.88	0.91	0.70
		
*FDXR*	1.24	0.63	**<0.001**			
				0.88	0.82	0.75
		
*POU2AF1*	1.06	1.44	**<0.001**			
				1.25	1.34	0.93
		
*WNT3*	0.96	0.80	0.089			
				0.91	0.60	**0.02**
		
*BAX*	1.06	0.95	**0.029**			
				1.02	0.96	0.28
		
*AEN*	1.17	0.90	**<0.001**			
				1.07	0.99	0.65
		
*EDA2R*	1.57	1.41	**0.019**			
				1.5	1.53	0.81
		
*MIR34AHG*	1.36	2.56	**0.018**			
				1.89	2.00	0.88
		
*PHLDA3*	1.29	0.89	**<0.001**			
				1.18	0.95	0.22
		

## Data Availability

The data presented in this study are available on request from the corresponding author. The data are not publicly available due to privacy and ethical restrictions.

## Abbreviations

The following abbreviations are used in this manuscript:

CT	Computed Tomography
qRT-PCR	Quick Real-Time Polymerase Chain Reaction
DLP	Dose–Length Product
LNT	Linear No-Threshold

## Appendix A

### Appendix A.1

**Table A1:** Differential gene expression (DGE, calibrated to pre-irradiation levels) for all target genes is presented for each patient. In addition, the individual dose–length product (DLP) and the estimated effective dose calculation are shown for each patient.

Patient Number	Differential Gene Expression Normalized to *PUM1*									Dose–Length Product [mGy·cm]	Estimated Effective Dose [mSv]
	Gene of Interest										
	*DDB2*	*FDXR*	*POU2AF1*	*WNT3*	*BAX*	*AEN*	*EDA2R*	*MIR34AHG*	*PHLDA3*		
1	1.72	1.91	0.97	0.96	1.11	1.26	1.57	4.17	2.30	1020	15.30
2	1.48	1.18	1.00	1.13	1.01	1.40	3.57	2.11	1.53	854	12.81
3	1.23	1.32	1.10	0.43	1.04	1.30	3.29	1.26	1.58	423	6.35
4	1.17	1.00	1.40	1.11	0.90	1.30	1.90	2.77	1.27	221	3.32
5	1.13	1.18	1.00	1.66	1.02	1.17	1.25	1.89	1.24	396	5.54
6	0.81	1.00	1.06	0.78	0.86	1.14	1.21	0.48	1.29	155	2.17
7	1.91	3.04	1.10	0.81	1.35	2.81	5.77	4.79	4.00	1725	24.15
8	1.09	1.57	0.82	0.58	0.95	1.52	2.12	3.05	1.44	533	8.00
9	1.04	1.34	1.14	1.68	1.20	1.02	1.23	2.18	1.29	259	3.89
10	0.73	0.61	1.35	0.68	0.89	0.77	1.11	2.53	0.98	784	11.76
11	0.88	0.96	1.48	0.99	0.91	1.18	3.38	6.09	1.11	1145	17.18
12	0.88	1.62	1.10	0.50	1.10	1.28	2.81	1.45	1.53	797	11.96
13	1.03	1.07	1.31	0.59	1.05	0.85	1.41	0.86	0.85	188	2.82
14	1.02	1.21	1.34	0.63	0.96	1.15	2.29	1.32	0.78	254	3.81
15	0.70	0.44	1.44	0.61	0.92	0.85	0.82	1.29	0.73	458	6.87
16	1.00	0.82	1.10	1.23	1.13	0.84	1.06	1.09	0.68	192	2.88
17	1.01	0.63	1.76	0.94	0.92	0.91	1.93	1.32	1.04	964	14.46
18	1.05	0.89	1.88	0.58	0.77	0.90	2.01	0.53	1.61	231	3.47
19	0.88	0.53	1.50	1.08	0.78	1.23	1.51	4.51	0.89	1501	22.52
20	0.62	0.66	1.66	0.42	0.84	0.92	1.64	0.71	1.03	729	10.94
21	1.18	1.63	0.82	1.09	1.16	1.39	1.48	5.53	1.88	1074	16.11
22	0.97	1.23	0.91	0.57	1.10	1.22	2.46	1.53	1.08	729	10.94
23	1.63	2.58	0.94	0.84	1.20	1.69	10.91	0.98	4.00	542	8.13
24	0.70	0.62	1.35	0.59	1.17	0.86	1.21	4.32	0.95	637	9.56
25	0.76	0.72	1.28	0.67	0.86	0.76	1.09	0.83	1.06	622	9.33
26	1.17	1.11	1.21	1.06	0.93	1.34	1.42	3.31	1.98	686	10.29
27	0.70	0.63	1.45	0.87	0.83	0.84	0.88	4.28	0.55	212	2.97
28	1.40	1.39	1.41	1.21	1.14	1.07	2.29	2.92	1.73	383	5.36
29	0.64	0.46	1.25	1.47	0.74	0.69	1.12	2.15	0.68	902	13.53
30	1.09	1.08	0.93	0.69	1.13	1.10	1.55	2.57	1.66	1312	19.68
31	0.69	0.49	1.16	0.39	0.92	0.65	1.13	2.68	0.63	794	11.91
32	1.12	1.47	1.25	0.91	1.25	1.28	2.24	0.22	3.32	397	5.96
33	0.85	1.07	1.76	0.57	1.08	1.34	2.96	1.20	1.50	1611	24.17
34	0.91	1.24	0.88	0.59	1.29	1.00	0.86	1.12	0.95	193	3.47
35	0.71	0.66	1.13	0.65	0.82	0.98	2.94	8.38	1.22	1072	16.08
36	0.82	0.71	1.74	0.96	0.94	0.99	1.41	6.04	0.86	726	10.89
37	0.99	0.96	0.88	0.68	0.82	0.91	1.45	0.60	1.15	422	6.33
38	0.95	0.75	1.36	0.79	1.02	0.90	1.86	13.24	1.12	731	10.97
39	0.81	0.62	1.58	0.84	0.82	0.90	1.06	0.96	0.84	128	1.79
40	1.30	1.47	1.00	1.76	1.01	1.28	1.39	0.53	1.18	514	7.71
41	0.92	0.75	0.98	1.10	0.72	1.05	1.79	1.09	0.75	67	0.94
42	0.71	0.59	1.23	0.82	0.98	0.87	1.49	2.45	1.06	888	13.32
43	0.63	0.51	1.24	0.99	0.86	0.75	1.53	1.40	0.72	409	1.27
44	0.68	0.64	1.30	0.80	0.88	0.88	1.56	8.23	1.01	509	7.64
45	0.86	0.85	2.00	0.87	1.22	0.96	1.08	6.27	1.18	156	2.18
46	0.47	0.43	1.46	0.58	0.79	0.80	0.35	9.08	0.62	146	2.04
47	0.68	0.43	1.41	0.54	0.70	0.92	1.56	2.75	0.42	179	2.51
48	0.85	0.74	1.50	0.85	1.00	1.16	1.61	4.57	0.71	357	5.00
49	1.09	1.42	1.06	1.55	1.25	1.09	1.08	0.23	1.18	263	3.95
50	0.69	0.48	2.18	1.07	0.94	0.88	1.03	10.17	0.59	115	1.61
51	0.86	1.50	0.91	0.96	1.06	1.08	1.53	0.76	1.32	394	5.91
52	0.60	0.39	1.47	0.42	0.61	0.70	1.53	0.26	0.60	378	5.67
53	0.73	0.65	1.24	0.60	0.95	1.06	1.61	2.55	0.89	622	9.33
54	0.80	0.82	1.04	0.84	1.11	1.24	1.36	0.92	0.54	695	10.43
55	1.15	0.88	2.11	1.63	1.46	1.57	1.01	1.19	1.39	134	1.88
56	0.69	0.56	1.26	0.55	1.02	0.86	0.72	4.96	0.67	492	7.38
57	0.42	0.41	0.90	0.32	0.69	0.65	0.82	2.00	0.79	117	1.64
58	0.99	0.89	1.74	1.04	1.48	1.51	1.81	0.38	1.42	362	5.07
59	0.91	0.73	2.02	1.25	1.14	1.44	1.32	1.04	1.56	664	9.96
60	0.70	1.09	1.16	1.01	1.16	0.97	1.50	1.53	1.24	253	3.80
61	-	-	-	-	-	-	-	-	-	324	4.54

### Appendix A.2

**Table A2:** Average γ-H2AX + 53BP1 foci counts per cell for the γ-H2AX patients pre- and post-irradiation, as well as resulting radiation-induced foci (RIF). In addition, the individual dose–length product (DLP) and the estimated effective dose calculation are shown for each patient.

Patient Number	Average γ-H2AX + 53BP1 Foci/Cell			Dose–Length Product [mGy·cm]	Estimated Effective Dose [mSv]
	Pre- Exposure	Post- Exposure	RIF		
43	0.63	0.46	−0.17	409	1.27
44	0.49	0.59	0.10	509	7.64
45	0.78	0.95	0.17	156	2.18
46	0.72	0.89	0.17	146	2.04
47	1.21	1.35	0.14	179	2.51
48	0.73	0.87	0.14	357	5.00
49	0.34	0.29	−0.05	263	3.95
50	0.28	0.58	0.30	115	1.61
51	0.63	0.45	−0.18	394	5.91
52	0.51	0.70	0.19	378	5.67
53	0.36	0.52	0.16	622	9.33
61	0.55	0.80	0.25	324	4.54

### Appendix A.3

**Table A3:** Indication and anatomic region for the CT scan of each patient, as well as conversion factor (k) for effective dose estimation and possible prior conditions.

Patient Number	Indication for Scan	Anatomic Region of Scan	Conversion Factor (k)	Prior Conditions
1	Assessment of vascular status	Neck/Thorax/Abdomen/Pelvis	0.015	
2	Staging	Neck/Thorax/Abdomen/Pelvis	0.015	pT1 squamous cell carcinoma
3	Planning Transcatheter aortic valve replacement	Thorax/Abdomen/Pelvis	0.015	Metastatic prostate cancer
4	Planning Transcatheter aortic valve replacement	Thorax/Abdomen/Pelvis	0.015	
5	Fracture rule-out	Thorax	0.014	
6	Left atrial appendage thrombus rule-out	Thorax	0.014	
7	Planning mitral valve replacement	Heart	0.014	
8	Indeterminate pancreatic mass	Thorax/Abdomen/Pelvis	0.015	
9	Planning Transcatheter aortic valve replacement	Neck/Thorax/Abdomen/Pelvis	0.015	
10	Assessment of vascular status	Whole Body	0.015	
11	Staging	Thorax/Abdomen/Pelvis	0.015	Status post-sigmoid carcinoma (without recurrence)
12	Planning Transcatheter aortic valve replacement	Thorax/Abdomen/Pelvis	0.015	Papillary renal cell carcinoma
13	Planning Transcatheter aortic valve replacement	Thorax/Abdomen/Pelvis	0.015	Prostate cancer
14	Planning Transcatheter aortic valve replacement	Thorax/Abdomen/Pelvis	0.015	
15	Assessment of vascular status	Thorax/Abdomen/Pelvis	0.015	
16	Planning Transcatheter aortic valve replacement	Thorax/Abdomen/Pelvis	0.015	Status post-adenocarcinoma of the distal esophagus
17	Assessment of vascular status	Neck/Thorax/Abdomen/Pelvis	0.015	
18	Planning Transcatheter aortic valve replacement	Thorax/Abdomen/Pelvis	0.015	Rheumatoid arthritis
19	Echinococcosis rule-out	Thorax/Abdomen/Pelvis	0.015	
20	Assessment of vascular status	Hals/Thorax/Abdomen/Pelvis	0.015	
21	Assessment of vascular status	Thorax/Abdomen/Pelvis	0.015	Mycotic aneurysm of the infrarenal aorta
22	Planning Transcatheter aortic valve replacement	Thorax/Abdomen/Pelvis	0.015	
23	Planning organ donation	Abdomen	0.015	
24	Superior mesenteric vein thrombosis follow-up	Abdomen/Pelvis	0.015	
25	Abdominal aortic aneurysm	Neck/Thorax/Abdomen/Pelvis	0.015	
26	Aortic Stent follow-up	Thorax/Abdomen/Pelvis	0.015	
27	Asthma	Thorax	0.014	
28	Porcelain aorta	Thorax	0.014	
29	Thoracic endovascular aortic repair follow-up	Neck/Thorax/Abdomen/Pelvis	0.015	
30	Recurrent incisional hernia	Abdomen/Pelvis	0.015	
31	Planning Transcatheter aortic valve replacement	Thorax/Abdomen/Pelvis	0.015	
32	Control spleen drainage	Abdomen	0.015	
33	Follow-up aortic dissection	Neck/Thorax/Abdomen/Pelvis	0.015	
34	Planning Transcatheter aortic valve replacement	Thorax/Abdomen/Pelvis	0.015	Status post-breast cancer (27 years ago, no recurrence)
35	Assessment of vascular status	Thorax/Abdomen/Pelvis	0.015	
36	Assessment of vascular status	Neck/Thorax/Abdomen/Pelvis	0.015	
37	Planning Transcatheter aortic valve replacement	Thorax/Abdomen/Pelvis	0.015	Status post-prostate cancer (without recurrence)
38	Abdominal aortic aneurysm	Neck/Thorax/Abdomen/Pelvis	0.015	
39	Left atrial appendage thrombus rule-out	Thorax	0.014	
40	Planning Transcatheter aortic valve replacement	Thorax/Abdomen/Pelvis	0.015	
41	Planning Transcatheter aortic valve replacement revision	Thorax	0.014	
42	Assessment of vascular status	Neck/Thorax/Abdomen/Pelvis	0.015	
43	Coronary bypass planning	Head/Neck	0.0031	
44	Planning inguinal hernia repair	Abdomen/Pelvis	0.015	
45	Internal carotid artery stenosis	Neck/Thorax	0.014	
46	Hereditary Hemorrhagic Telangiectasia	Thorax	0.014	
47	Left atrial appendage thrombus rule-out	Thorax	0.014	
48	Aneurysma Aortenbulbus	Thorax	0.014	
49	Planning Transcatheter aortic valve replacement	Thorax/Abdomen/Pelvis	0.015	
50	Left atrial appendage thrombus rule-out	Thorax	0.014	
51	Thrombangitis	Thorax/Abdomen	0.015	
52	Planning Transcatheter aortic valve replacement	Neck/Thorax/Abdomen/Pelvis	0.015	
53	Assessment of vascular status	Thorax/Abdomen/Pelvis	0.015	
54	Assessment of vascular status	Whole Body	0.015	
55	Left atrial appendage thrombus rule-out	Thorax	0.014	
56	Assessment of vascular status	Thorax/Abdomen/Pelvis	0.015	
57	Coronary status and calcium scoring	Heart	0.014	
58	Left atrial appendage thrombus rule-out	Thorax	0.014	
59	Assessment of vascular status	Neck/Thorax/Abdomen/Pelvis	0.015	
60	Planning Transcatheter aortic valve replacement	Thorax/Abdomen/Pelvis	0.015	
61	Left atrial appendage thrombus rule-out	Thorax	0.014	
